# The Characterization and Modification of a Novel Bifunctional and Robust Alginate Lyase Derived from *Marinimicrobium* sp. H1

**DOI:** 10.3390/md17100545

**Published:** 2019-09-23

**Authors:** Junjun Yan, Peng Chen, Yan Zeng, Yan Men, Shicheng Mu, Yueming Zhu, Yefu Chen, Yuanxia Sun

**Affiliations:** 1 College of Bioengineering, Tianjin University of Science and Technology, Tianjin 300457, China; yanjj@tib.cas.cn (J.Y.); mushc@tib.cas.cn (S.M.); yfchen@tust.edu.cn (Y.C.); 2 National Engineering Laboratory for Industrial Enzymes, Tianjin Institute of Industrial Biotechnology, Chinese Academy of Sciences, Tianjin 300308, China; chen_p@tib.cas.cn (P.C.); zeng_y@tib.cas.cn (Y.Z.); men_y@tib.cas.cn (Y.M.); Sun_yx@tib.cas.cn (Y.S.)

**Keywords:** alginate lyase, PL7 family, alginate oligosaccharides, bifunctional, robust enzyme

## Abstract

Alginase lyase is an important enzyme for the preparation of alginate oligosaccharides (AOS), that possess special biological activities and is widely used in various fields, such as medicine, food, and chemical industry. In this study, a novel bifunctional alginate lyase (AlgH) belonging to the PL7 family was screened and characterized. The AlgH exhibited the highest activity at 45 °C and pH 10.0, and was an alkaline enzyme that was stable at pH 6.0–10.0. The enzyme showed no significant dependence on metal ions, and exhibited unchanged activity at high concentration of NaCl. To determine the function of non-catalytic domains in the multi-domain enzyme, the recombinant AlgH-I containing only the catalysis domain and AlgH-II containing the catalysis domain and the carbohydrate binding module (CBM) domain were constructed and characterized. The results showed that the activity and thermostability of the reconstructed enzymes were significantly improved by deletion of the F5/8 type C domain. On the other hand, the substrate specificity and the mode of action of the reconstructed enzymes showed no change. Alginate could be completely degraded by the full-length and modified enzymes, and the main end-products were alginate disaccharide, trisaccharide, and tetrasaccharide. Due to the thermo and pH-stability, salt-tolerance, and bifunctionality, the modified alginate lyase was a robust enzyme which could be applied in industrial production of AOS.

## 1. Introduction

Brown algae is a kind of macroalgae and is wildly distributed in marine ecosystems. Alginate from the cell walls of brown algae is a linear polysaccharide [1], which consists of β-d-mannuronate (M) and α-l-guluronate (G) [2]. The monomers appears in poly-β-d-mannuronate (polyM), poly-α-l-guluronate (polyG), and the heteropolymer (polyMG) [3,4]. The arrangement order and distribution ratio of the above three blocks in alginate have an important influence on its physical and chemical properties [5]. Alginate is highly viscous and gelatinous, and is non-toxic to organisms, so it has been widely used in many fields, such as food, medicine, and cosmetics [6,7].

Alginate oligosaccharide (AOS), the degradation product of alginate, has attracted much attention due to its diverse functional activities and application value. It has been gradually applied in green agriculture, food, medicine, and other fields [8]. As reported, AOS has been shown to be a bifidogenic factor that promotes plant growth and enhances the growth of human endothelial cells and keratinocytes [9]. AOS can be used in medicine and healthy food because it possesses special biological activities, such as anti-tumor, anti-inflammatory, anti-coagulation, anti-oxidation, immune regulation, and blood lipid and blood sugar lowering effects [10,11]. In addition, protoplast fragments produced during the degradation of alginate can be exploited as feed for animals [12].

Alginate lyase that cleaves alginate glycosidic bonds by β-elimination mechanism is an efficient tool for the production of AOS [13,14,15]. Compared to acid or base hydrolysis, enzymatic degradation is mild, easy to control, efficient, and environmentally-friendly [16]. Hence, various alginate lyases from marine and terrestrial bacteria, molluscs, and algae plants have been screened [17,18], and more than 100 alginate lyases have been characterized [19]. Alginate lyases belong to polysaccharide lyase (PL) families, and are classified into the PL5, 6, 7, 14, 15, 17, and 18 families. According to the mode of action of degrading alginate, alginate lyase can be divided into endo-type and exo-type [20]. The endo-type mainly hydrolyzes glycosidic bonds by β-elimination reaction to produce unsaturated disaccharides, trisaccharides and tetrasaccharides as main products, while the exo-type can hydrolyze oligosaccharides to form monosaccharides [21,22]. According to its substrate specificity, alginate lyase can be divided into polyM lyase, polyG lyase, and bifunctional lyase which can degrade polyM and polyG [23,24]. The bifunctional lyase can degrade alginate more efficiently and completely, but the number of this kind is relatively small [25,26]. Moreover, due to the low activity and stability, few of the reported alginate lyases have been applied in industrial production [27].

In this study, a novel bifunctional alginate lyase (AlgH) from *Marinimicrobium* sp. H1 was cloned and expressed in *Escherichia coli*, and the enzymatic properties of the enzyme were characterized in detail. The carbohydrate binding module (CBM) and F5/8 type C domain was fully or partially truncated to construct the recombinant AlgH-I and AlgH-II, which showed improved activity and thermostability. Analysis of the degradation products revealed that the AlgH could completely degrade alginate to form di, tri, and tetra-saccharides as the main end-products. The experimental results suggested that the novel AlgH has great advantages and potential in the industrial production of AOS.

## 2. Results and Discussions

### 2.1. Sequence Analysis of AlgH

**Figure 1:**
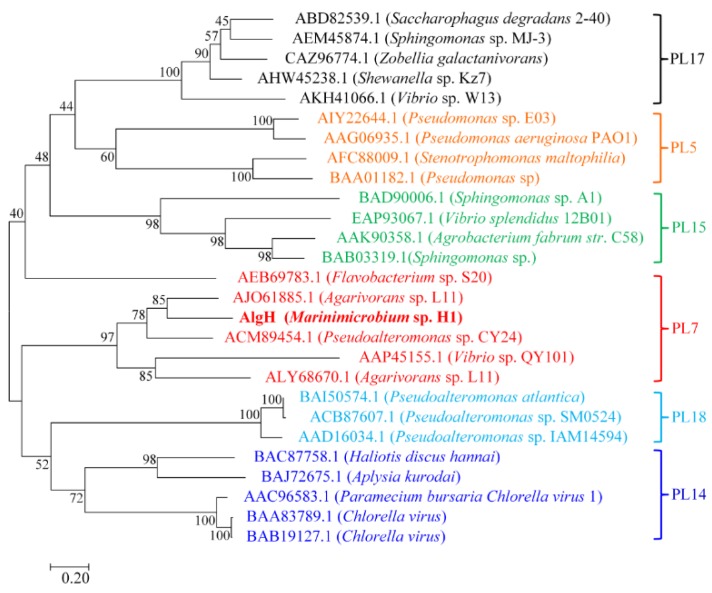
Phylogenetic tree of AlyH with other reported alginate lyases. Evolutionary analysis was inferred using the Neighbor-Joining method in MEGA7.

The alginate lyase AlgH (GeneBank number MN257508) was obtained from a marine bacterium, *Marinimicrobium* sp. H1. The gene of AlgH consists of 1731 base pairs and encodes a protein containing 576 amino acid residues. The calculated molecular weight of AlgH was 61.3 kDa. The AlgH belongs to the polysaccharide lyases family according to Carbohydrate-Active Enzymes (CAZy) Database. A phylogenetic tree containing various alginate lyases was generated using the Neighborhood-joining method (Figure 1), and it indicated that AlgH belonged to PL7 family.

**Figure 2:**
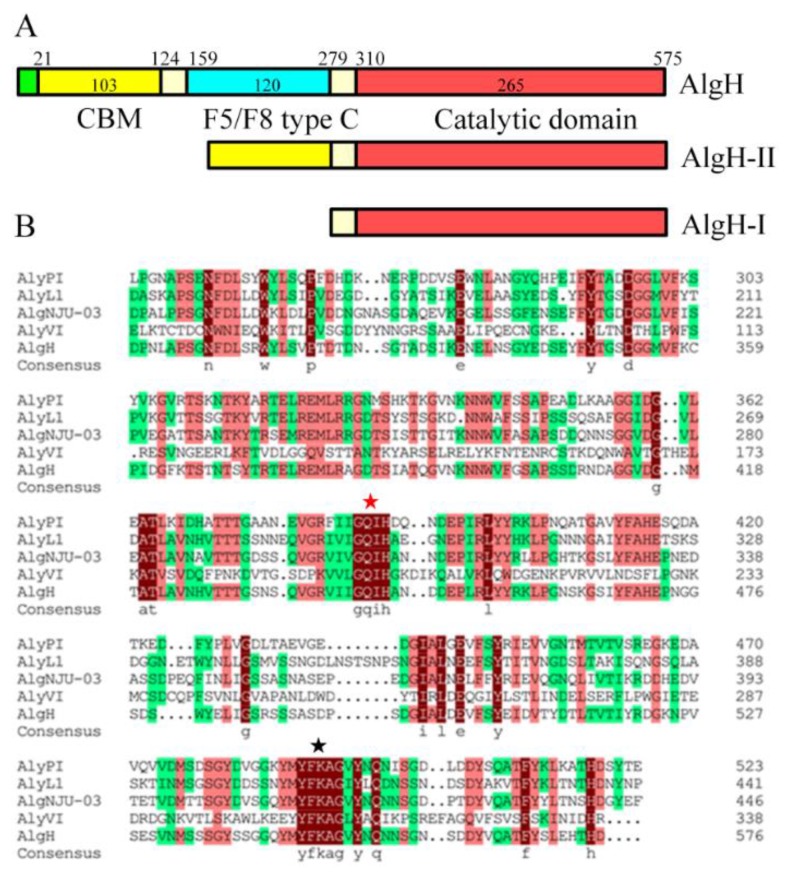
Sequence analysis of AlgH from *Marinimicrobium* sp. H1. (**A**) The domain composition of AlgH. The green, yellow, blue, and red colors correspond to signal peptide, carbohydrate binding module (CBM), F5/8 type C, and catalytic domain, respectively. (**B**) Amino acid alignment of catalytic active region sequence of AlgH to other reported PL7 alginate lyases. AlgH from *Marinimicrobium* sp. H1 in this study, AlyPI (ACM89454.1) from *Pseudoalteromonas* sp. CY24 [28], AlyL1 (AJO61885.1) from *Agarivorans* sp. L11 [29], AlgNJU-03 (ASA33933.1) from *Vibrio* sp. NJU-03 [30] and the conserved regions of QIH and YFKAG are boxed.

The AlgH was predicted as a secretion protein within the Sec pathway using SignalP-4.1 server, and its signal peptide comprised the first 21 amino acid residues. The enzyme was a multi-domain alginate lyase, which contained a catalytic domain, a carbohydrate binding module (CBM), and an F5/8 type C domain (Figure 2). In addition, multiple sequence alignment of the catalytic domain of AlgH and other alginate lyases of the PL7 family indicated that the AlgH contained two conserved regions of QIH and YFKAG.

### 2.2. Biochemical Characteristics of AlgH

**Figure 3:**
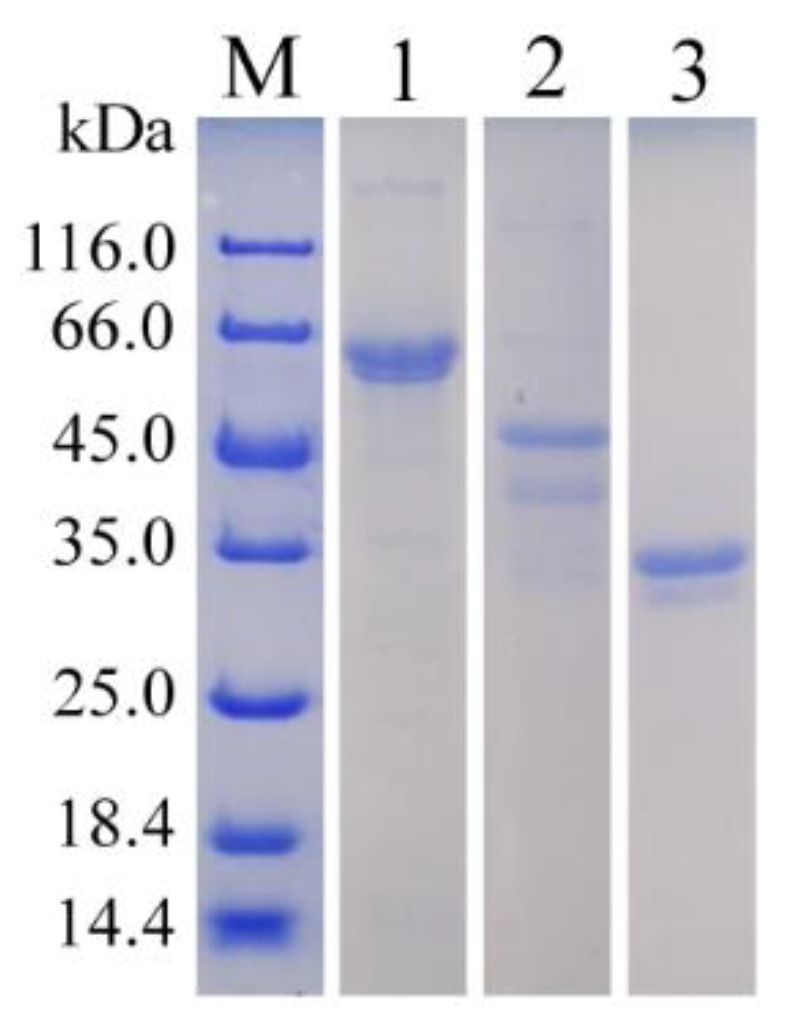
The SDS-PAGE analysis of the purified AlgH, AlgH-I, and AlgH-II. Lane M, the protein molecular weight standard; Lane 1, purified AlgH; Lane 2, purified AlgH-II; Lane 3, purified AlgH-I.

AlgH was expressed in *E. coli* BL21 (DE3) strain using the expression vector of pET-21a. The enzyme was purified by nickel column affinity chromatography and ultrafiltered using a 30 kDa ultrafiltration tube. The purified enzyme showed a single main band whose molecular weight was 61.3 kDa in SDS-PAGE (Figure 3). 

**Figure 4:**
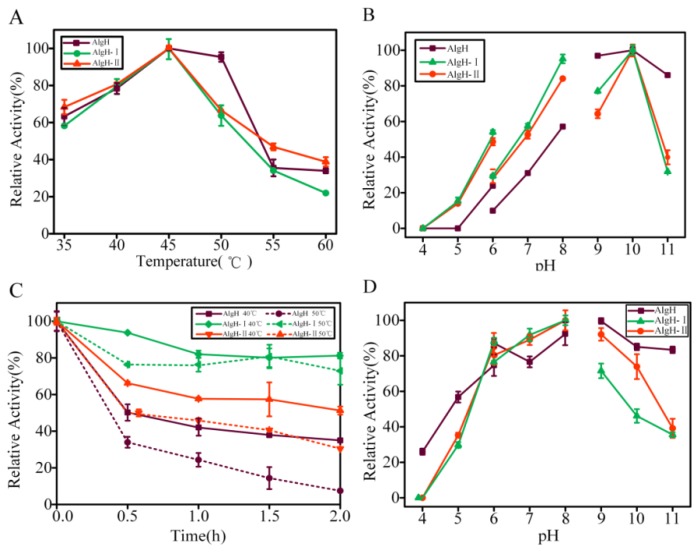
Biochemical characteristics of three recombinant alginate lyases. (**A**) The optimal temperatures of AlgH (purple), AlgH-I (green), and AlgH-II (red). (**B**) The optimal pHs of AlgH (purple), AlgH-I (green), and AlgH-II (red). (**C**) The thermal stabilities of AlgH (purple), AlgH-I (green), and AlgH-II (red). Solid and dashed lines correspond to incubation temperatures of 40 and 50 °C, respectively. (**D**) The pH stabilities of AlgH (purple), AlgH-I (green), and AlgH-II (red).

The effects of temperature and pH on the AlgH were determined using sodium alginate as the substrate. The results indicated that the optimal temperature for the enzymes was 45 °C (Figure 4A). The optimal pH of AlgH reached 10.0 (Figure 4B), which suggested that the enzyme was an alkaliphilic alginate lyase. As reported, most alginate lyases presented their highest activity at 7.0–8.5. Only the alginate lyase from *Agarivorans* sp. (A1m) and the Chlorella virus-encoded CL2 showed peak activities at pH 10.0 and 10.5, respectively [31,32]. 

**Figure 5:**
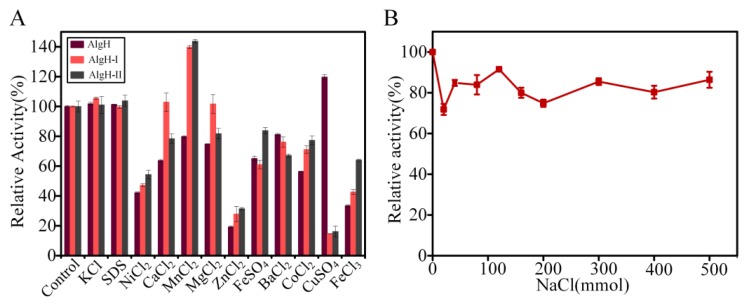
The effects of various metal ions on the activity of the recombinant alginate lyases. The enzymatic activity without the addition of metal ions was defined as 100%. Dark gray, red, and light gray represent AlgH, AlgH-I, and AlgH-II, respectively.

Moreover, the effect of metal ions on the activity of AlgH is shown in Figure 5A. The results suggested that the activity of AlgH was independent on metal ions, and obviously inhibited by Ni^2+^, Zn^2+^, and Fe^3+^. In addition, the AlgH was a salt-tolerant enzyme, which could maintain activity at a high concentration (500 mM) of NaCl (Figure 5B). Therefore, this AlgH could be used in the degradation of substrates with high salt content; e.g., brown algae from marine environments could be directly degraded by AlgH without desalting.

### 2.3. The Effect of the Non-Catalytic Domain of AlgH

To determine the function of non-catalytic domains, the CBM and F5/8 type C domain were deleted to obtain AlgH-I, while the CBM domain was fused with the catalytic domain to obtain AlgH-II (Figure 2A). AlgH-I and AlgH-II were expressed in *E. coli* BL21 (DE3) and purified. The results of SDS-PAGE showed the reconstructed enzymes presented high purity and correct molecular weight (Figure 3). 

The biochemical characteristics of AlgH-I and AlgH-II were determined using the same method as the full-length AlgH. Surprisingly, the thermostabilities of the truncated enzymes were significantly enhanced, especially that of AlgH-I, whose residual activity remained at 80% or 75% after incubation for 2 h at 40 °C or 50 °C, respectively (Figure 4C). In contrast, the residual activity of AlgH only remained at about 10% after incubation at 50 °C for 2 h. Furthermore, by the modification of protein domains, the activities of the AlgH-I and AlgH-II were 2.1-fold and 1.3-fold greater than that of AlgH. The specific activity of the purified AlgH-I was 5510 U/mg in the optimal conditions. It was indicated that the predicted CBM and F5/8 type C domain might affect the structure of the catalytic domain and decrease the thermostability and activity of the enzyme. 

On the contrary, the non-catalytic domain of most reported alginate lyases played an important role in enzymatic activity and thermal stability. For instance, the removal of the N-terminal CBM13 of AlyL2 from *Agarivorans* sp. L11 decreased the catalytic efficiency and half-life of the enzyme [33], and the deletion of the F5/8 type C domain of Aly5 from *Flammeovirga* sp. MY04 showed the similar effect [19]. The activity and stability of an enzyme are crucial factors to determine whether the enzyme could be used in industrial production. The improvement of thermostability and activity of AlgH-I makes it a good candidate for industrial application.

For pH stability, although the removal of the non-catalytic domains might slightly decrease the enzyme stability in strongly alkaline environment (pH > 10.0), the AlgH-I was still pH-stable and could maintain high activity over a wide range of pHs (6.0–9.0) (Figure 4D). Moreover, the activities of AlgH-I and AlgH-II in Tris-HCl buffer (pH 8.0) were as high as those in Glycine-NaOH buffer (pH 10.0). Hence, less base is required to adjust the pH of the degradation reaction, and the conditions of the enzymatic process are mild and environmentally-friendly.

### 2.4. Substrate Specificity and Enzymatic Kinetics of the Recombinant Alginate Lyases

**Figure 6:**
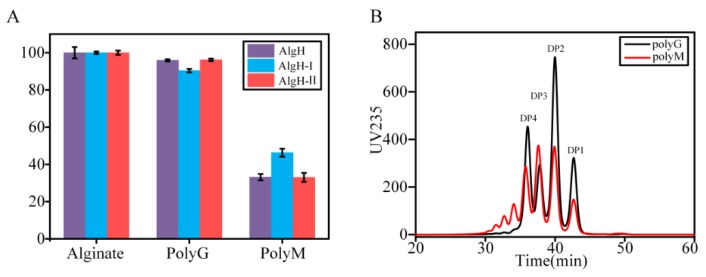
Substrate preference of the recombinant alginate lyases. (**A**) The relative activities towards alginate, polyG, and polyM. (**B**) HPLC analyses of the degradation products of polyG and polyM.

To study the substrate specificity of the enzyme, we used 13 polysaccharides as substrates for the activity assay. The results indicated that the enzyme only exhibited activity towards the alginate series of substrates. The AlgH showed high activity toward sodium alginate, polyG, and polyM (Figure 6A), which suggested the enzyme was a bifunctional alginate lyase. The relative activity towards polyG was higher than that towards polyM, and the degradation products of polyG had smaller degrees of polymerization than those of polyM (Figure 6B). The substrate specificity was almost unchanged by the removal of the CBM and F5/8 type C domains.

Until now, nineteen bifunctional alginate lyases have been characterized and reported, and they belong to PL6, 7, 15, 17, and 18 families. Thirteen of them belong to PL7 family, such as AlgM4 from *Vibrio weizhoudaoensis* M0101 [8], AlyH from *Isoptericola halotolerans* NJ-05 [27], and AlgNJ-04 from *Vibrio* sp. NJ-04 [4]. However, the activities of most bifunctional alginate lyases are very low (Table 1), which limits the industrial application of them. The modified AlgH-I in this study presented high activity (5510 U/mg), which was a little lower than that of AlgNJU-03 from *Vibrio* sp. NJU-03. The content of the recombinant AlgH-I was about 11% of the total protein of host strain, and the yield of the protein was about 167.2 mg/L. Meanwhile, the thermostability of the AlgH-I was significantly improved by deletion the CBM and F5/8 type C domains. Furthermore, the bifunctional AlgH-I in this study could degrade both polyG and polyM, which makes the degradation of alginate more efficient and complete. Therefore, the novel alginate lyase with high activity, strong thermo and pH-stability and salt-tolerance was a robust enzyme that might be used in industrial application.

The kinetic parameters of AlgH, AlgH-I, and AlgH-II were determined according to nonlinear regression analysis. As shown in Table 2, the K_m_ values of the alginate lyases towards sodium alginate and PolyG were lower than that towards polyM, which indicated that the enzymes have higher affinity for sodium alginate and polyG. The K_cat_/K_m_ values of the alginate lyases for different substrates were sodium alginate > polyG > polyM, which suggested that the enzymes exhibited highest catalytic efficiency for sodium alginate. Moreover, the modified enzymes, AlgH-I and AlgH-II, showed similar K_m_ values to the wild-type enzyme, which indicated that the removal of the CBM and F5/8 type C domains could not affect the substrate specificity of the enzyme.

### 2.5. Degradation Mode and Product Analysis of the Alginate Lyase

**Figure 7:**
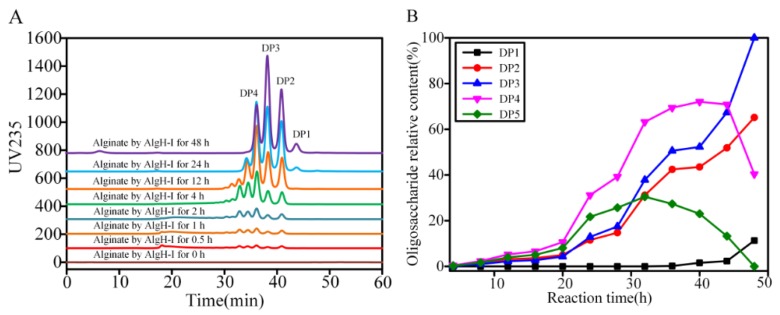
The analyses of degradation products produced by AlgH-I. (**A**) Time course analysis of oligosaccharide products of alginate. (**B**) Relative contents of oligosaccharides. HPLC analyses were performed using a Superdex peptide 10/300 GL column monitored at a wavelength of 235 nm. DP1, DP2, DP3, DP4, and DP5 represent mono, di, tri, tetra, and penta-saccharide, respectively.

The degradation products of AlgH-I were analyzed by a gel filtration column using HPLC. The mode of action was inferred from the change in the type and amount of oligosaccharide produced by the time of degradation of the substrate by AlgH-I. In the initial stage of degradation, the alginate was rapidly degraded and dispersed, and the viscosity of the solution was rapidly decreased. Oligosaccharides with higher degrees of polymerization were produced first. After degrading for a period of time, the intermediate products, disaccharides, trisaccharides, and tetrasaccharides were the main products (Figure 7A). During the degradation process, disaccharides and trisaccharidse continued to accumulate, and most of the pentasaccharide and some of the tetrasaccharide continued to degrade into disaccharides and trisaccharides (Figure 7B). The results indicated that the enzyme was an endo-type alginate lyase. Based on the action mode of AlgH-I, a controlled degradation method was established to obtain AOS with different degrees of polymerization by controlling the enzyme dose and reaction time. The degradation products of alginate by the AlgH and AlgH-II were also determined, and the results showed that there was no significant difference between the full-length and truncated enzymes. It was suggested that the CBM and F5/8 type C domains were not concerned with the degradation model of the enzyme. 

### 2.6. ESI-MS and ^1^H-NMR Spectral Analyses of Degradation Products

**Figure 8:**
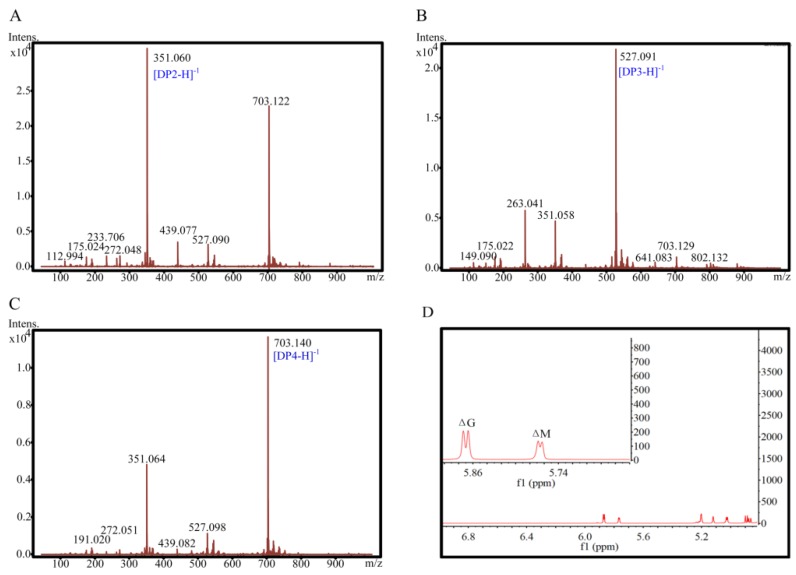
ESI-MS and ^1^H-NMR spectral analysis of the purified degradation products of alginate. (**A**–**C**) represent the ESI-MS results of di, tri, and tetra-saccharide, respectively. (**D**) ^1^H-NMR spectra of the purified disaccharide (600-MHz).

The degradation products of alginate were separated and purified, and then the main products were identified as disaccharides, trisaccharides, and tetrasaccharides by ESI-MS (Figure 8). The purified disaccharide was further analyzed by ^1^H-NMR spectroscopy. The signal at 5.86–5.88 ppm indicated that the N-terminal residue of the oligosaccharide was ΔG, while the signal at 5.76–5.78 ppm indicates that the N-terminal residue of the oligosaccharide was ΔM. Therefore, the disaccharide is an oligosaccharide containing a unit of ΔG, ΔM.

## 3. Materials and Methods

### 3.1. Cloning and Expression of Recombinant Alginate Lyases

The Marinimicrobium sp. Strain H1 was isolated from rotten kelp samples on the coast in Weihai City, Shandong province, China. The genome of the strain was extracted using TIANamp Bacteria DNA Kit (Tiangen, Beijing, China). The gene fragments of AlgH, AlgH-I, and AlgH-II was cloned by polymerase chain reaction (PCR) using the primers listed in Table 3. The PCR products were purified, digested with Nde I and Xho I, and ligated to the vector pET-21a. The recombined plasmids were transformed into the *E. coli* BL21 (DE3) strain for heterologous expression. The strains were inoculated in LB medium containing 100 μg/mL ampicillin at 37 °C and 200 rpm. When the OD_600_ reached to 0.6–0.8, the heterologous proteins were induced by 1 mM isopropyl β-D-Thiogalactoside (IPTG) at 16 °C and 200 rpm for 24 h.

### 3.2. The Purification of Recombinant Alginate Lyases

The bacterial cells after induction were centrifuged at 6000 rpm for 10 min. The harvested cells were disrupted by sonication treatment in 50 mM Tris-HCl buffer (pH 7.0). The supernatants were obtained by centrifuging at 20,000 rpm for 30 min. The heterologous proteins were purified by affinity chromatography using a column packed with NTA-Ni resin. The binding buffer (50 mM Tris-HCl, 500 mM NaCl, 20 mM imidazole, pH 7.0) was used to equilibrate the resin and remove the host protein, while the elution buffer (50 mM Tris-HCl, 500 mM NaCl, 500 mM imidazole, pH 7.0) was used to obtain the heterologous protein. The purified alginate lyases were determined by dodecyl polyacrylamide gel electrophoresis (SDS-PAGE), and the protein concentrations were tested using the BCA(Solarbio, Beijing, China) protein assay.

### 3.3. Enzymatic Activity Assay

The appropriately diluted enzyme of 50 μL was added into the 1 mL reaction solution containing 10 g/L sodium alginate as the substrate. The reaction was carried out at 45 °C for 30 min, and was quenched with 20 μL of 10 M NaOH. The absorbance of product was determined at 235 nm. One unit of enzyme activity was defined as the amount of the enzyme required to increase the absorbance at 235 nm by 0.1 per minute.

### 3.4. Determination of the Substrate Specificities and Enzymatic Kinetics of Recombinant Alginate Lyases

Thirteen polysaccharides were used as the substrates to determine the substrate specificities of AlgH, AlgH-I, and AlgH-II. The polysaccharides were pectin, carrageenan, hyaluronic acid, agar, starch, sodium carboxymethylcellulose, inulin, chitosan, agarose, xylan, sodium alginate, polyG, and polyM. The concentration of the substrate was 10 g/L, and then the enzyme activity was measured by the DNS method. Different substrates were incubated with the diluted pure enzyme at 45 °C for 20 min, the reaction was stopped by adding DNS, boiled at 100 °C for 5 min, and the absorbance was measured at 540 nm. [36].

The kinetic parameters of the enzymes were determined by measuring the activity of the pure enzymes at different concentrations (1–12 mg/mL) for different substrates (sodium alginate, PolyG, and PolyM). The nonlinear regression equation was used to fit and calculate V_max_ and K_m_.

### 3.5. The Biochemical Characterization of Recombinant Alginate Lyases

The optimum temperatures of AlgH, AlgH-I, and AlgH-II were tested a the temperature range from 35 °C to 60 °C. The appropriately diluted enzyme solution of 100 μL was added into the 1.9 mL reaction solution containing 10 g/L sodium alginate as the substrate. The reaction was carried out at different temperatures for 20 min. The enzyme activity was measured by DNS method. For temperature stability test, the diluted enzymes were incubated at 40 and 50 °C for 2 h, and the residual activity was measured as described above. To determine the effect of pH on alginate lyases, the reaction was carried out in CH_3_COOH-CH_3_COONa buffer (100 mM, pH 4.0–6.0), Tris-HCl buffer (100 mM, pH 6.0–8.0), and Glycine-NaOH buffer (100 mM, pH 9.0–11.0). For pH stability tests, the enzymes were incubated in the buffers at 4 °C for 12 h, and the residual enzymatic activities were measured. 

To determine the effect of metal ions on the activity of AlgH, AlgH-I, and AlgH-II, the enzymes were incubated with various metal ions at the final concentration of 5 mM at 4 °C for 12 h, and then the enzyme activity was measured by the DNS method as described above. The enzyme activity without metal ions was used as the control.

### 3.6. HPLC Analysis of Degradation Products

To analyze the degradation products of alginate by the alginate lyases, the purified enzymes were used to degrade sodium alginate at room temperature for 48 h. The samples at different time intervals were boiled for 5 min, centrifuged at 14,000 rpm for 5 min, and filtered with 0.22 μm sterile filters. The oligosaccharide products were gel filtered using a Superdex peptide 10/300 GL column (GE Healthcare, Florence, SC, USA) and monitored at 235 nm using a UV detector on the high-performance liquid chromatography (HPLC) system (Agilent Technologies, Santa Clara, CA, USA). The mobile phase was 0.2 M NH_4_HCO_3_, and the flow rate was 0.4 mL/min.

### 3.7. ESI-MS and ^1^H-NMR Spectral Analysis of Degradation Products

For the preparation of the alginate digests, excess enzyme was used to completely degrade alginate (10 g/L). The oligosaccharides were separated by gel filtration by the same method described above. Each fraction was collected and repeatedly freeze-dried to remove excess NH_4_HCO_3_ for ESI-MS (Bruker-micrOTOF, Billerica, MA, USA) analysis. The purified disaccharide product was dissolved in deuterium oxide with a final concentration of 20 mg/mL, and analyzed by ^1^H-NMR spectroscopy (Bruker AVANCE III 600 m/z, Billerica, MA, USA).

## 4. Conclusions

In this study, a novel bifunctional alginate lyase (AlgH) from *Marinimicrobium* sp. H1 was characterized and modified by domain reconstruction. The activity and thermostability of the truncated AlgH-I were both significantly enhanced by removing the CBM and F5/8 type C domains, while the substrate specificity and degradation model of the enzyme were hardly changed by domain deletion. The enzyme could completely degrade alginate to produce alginate disaccharide, trisaccharide, and tetrasaccharide as the final main products. Furthermore, the enzyme showed no significant dependence on metal ions, and exhibited unchanged activity at high concentrations of NaCl. The properties of the enzyme indicate that the novel alginate lyase is a robust enzyme with high activity, thermal and pH-stability, and salt-tolerance, which makes the conditions of enzymatic reaction more flexible and variable. Hence, the novel alginate lyase might be a potential industrial enzyme for the efficient production of AOS.

## Figures and Tables

**Table 1:** Summarization of the reported bifunctional alginate lyases.

	Enzyme	Organism	Optimal pH	Optimal Temperature (°C)	Activity (U/mg)	Reference
PL6	KJ-2	*Stenotrophomas Maltophilia*	8.0	40	N.D	[34]
PL7	AlgH-I	*Marinimicrobium koreense* H1	10.0	45	5510	This study
	Aly7B_Wf	*Fucanilytica* *Vibrio*	8.5	40	23.24*	[13]
	AlgM4	*Vibrio weizhoudaoensis* M0101	8.5	30	4638^#^	[8]
	rAlgSV1-PL7	*Shewanella Species* YH1	8.0	45	N.D	[35]
	AlyA	*Isoptericola Halotolerans* NJ-05	7.5	55	7984 ^#^	[27]
	AlgNJU-03	*Vibrio* sp. NJU-03	7.0	30	6468.9	[30]
	AlgNJ-04	*Vibrio* sp. NJ-04	7.0	40	2416	[4]
	AlyH1	*Vibrio furnissii* H1	7.5	40	2.40 *	[36]
	AlgMsp	*Microbulbifer* sp. 6532A	8.0	50	N.D	[37]
	AlyL1	*Agarivorans* sp. L11	8.6	40	1370	[29]
	AlyPI	*Pseudoalteromonas* sp. CY24	7.0	40	N.D	[28]
	A1-II’	*Sphingomonas* sp. Strain A1	7.5	40	2.89	[38]
	AlyVI	*Vibrio* sp. QY101	7.5	40	N.D	[39]
PL15	A1-IV’	*Sphingomonas* sp. A1	8.5	50	12.1	[40]
PL17	Alg17c	*Saccharophagus Degredans*	7.5	30	N.D	[41]
	Oal17A	*Vibrio* sp. W13	7.0	30	2.11	[42]
	AlgL	*Sphingomonas* sp. MJ-3	6.5	50	N.D	[43]
PL18	Aly-SJ02	*Pseudoalteromonas* sp. SM0524	N.D	N.D	N.D	[44]

Note: “*” represents the amount of reducing sugar released using the 3,5-dinitrosalicylic acid (DNS) method to determine alginate lyase activity. The enzyme activity measurement method not marked with “*” is the 235 nm absorbance method and one unit was defined as the amount of enzyme required to increase the absorbance at 235 nm by 0.1 per min, while “^#^” represents one unit, defined as the amount of enzyme required to increase the absorbance at 235 nm by 0.01 per min.

**Table 2:** Enzyme kinetic parameters of recombinase towards sodium alginate, polyG and polyM.

Substrate	Enzyme	*V_max_* (U∙mg of protein^−1^)	*K_m_* (mg∙mL^−1^)	*k_cat_* (s^−1^)	*k_cat_/K_m_* (mg^−1^∙mL∙s^−1^)
Sodium Alginate	AlgH	224.6 ± 33.6	6.6 ± 2.2	260.6 ± 36.2	39.5 ± 5.2
	AlgH-I	450.5 ± 82.1	6.9 ± 2.6	262.6 ± 41.5	38.1 ± 4.9
	AlgH-II	340.2 ± 70.3	7.7 ± 3.3	255.5 ± 32.9	33.2 ± 4.1
PloyG	AlgH	146.6 ± 15.6	7.6 ± 1.6	155.7 ± 17.1	20.5 ± 2.6
	AlgH-I	277.8 ± 21.3	7.3 ± 1.5	162.1 ± 16.8	22.2 ± 2.4
	AlgH-II	199.7 ± 18.5	7.5 ± 1.2	149.8 ± 13.5	19.9 ± 1.8
PloyM	AlgH	62.6 ± 8.8	9.1 ± 2.4	66.8 ± 6.7	7.3 ± 0.5
	AlgH-I	106.8 ± 17.1	8.1 ± 2.5	62.3 ± 5.6	7.6 ± 0.3
	AlgH-II	78.4 ± 9.8	7.9 ± 1.9	58.8 ± 4.9	7.4 ± 0.2

**Table 3:** Primers used for amplification of AlgH, AlgH-I, and AlgH-II.

Primer Name	Nucleotide Sequence (5′to 3′)
AlgHf	AAGAAGGAGATATACATATGAAAATCAACAGGTTACTTCCTTTC
AlgHr	TGGTGGTGGTGGTGCTCGAGATCGTGGGTGTGCTCAAGGG
AlgH-If	AAGAAGGAGATATACATATGCAGGTGGGTTGCGGCGATTTTGCCG
AlgH-Ir	AGTGGTGGTGGTGGTGGTGCTCGAGATCGTGGGTGTGCTCAAGGG
AlgH-IIf	AAGAAGGAGATATACATATGAGCTTGACCAACCCGGGCTTTG
AlgH-IIr	AGTGGTGGTGGTGGTGGTGCTCGAGATCGTGGGTGTGCTCAAGGG
FU-F	CGCGAGCAGCGGCGAGGTGTTCGCACAGGTGGGTTGCGGCGATTT
FU-R	CGGCAAAATCGCCGCAACCCACCTGTGCGAACACCTCGCCGCTGCT
